# Supplementary material for: Are carbon emissions trading and green financial instruments synergistic? -Comprehensive quantitative research based on content analysis
Source: PLoS One. 2024 Mar 7;19(3):e0298601. doi: 10.1371/journal.pone.0298601 (PMC10919601; doi:10.1371/journal.pone.0298601)
Supplement: S1 Data — (ZIP) [file pone.0298601.s001.zip › Data/地级市Z_F工作报告.zip!/║┌┴·╜¡/║┌║╙╩╨/└┤╫╘╣1⁄2╓┌║┼í╛┬φ┐╦╩2╛▌═°í┐_2013.docx]

各位代表：

现在，我代表市人民政府向大会作工作报告，请予审议，并请列席会议的同志提出宝贵意见。

一、2012年工作回顾

在省委省政府和市委正确领导下，我们紧紧依靠全市人民，认真践行科学发展观，实施“三大战略”，打好“四张牌”，发育“五大产业”，建设“一都三地”魅力黑河，圆满完成五届人大一次会议确定的任务目标。预计全市生产总值360亿元，增长13%。全社会固定资产投资213亿元，增长35%，其中工业固定资产投资增长60%。社会消费品零售总额73.6亿元，增长15%。公共财政收入21亿元，按可比口径增长20%。对外贸易进出口总值37亿美元，增长15%。城镇居民人均可支配收入16500元，农村居民人均纯收入9700元，均增长12%。万元地区生产总值综合能耗下降3.3%。

（一）把握关键抢先机，未来发展再添助力。坚持跳出黑河看黑河，把黑河放在全国全省大局中谋划，找准制约经济社会发展的关键，赢得支持。政策争取实现新突破。经过不懈努力，成功纳入国家“三大规划、两大试点”。中国东北地区面向东北亚区域开放规划、黑龙江和内蒙古东部沿边开发开放规划、兴边富民行动规划，把黑河定位为东北北部沿边生态宜居城市和中俄友好示范城市，在界江桥梁、边境经济合作区和俄电贸易加工区建设等方面给予重点扶持，将完善口岸功能，优化产业布局，提升我市在全国沿边开放中的战略地位。市区、北安、逊克纳入全国发展改革试点，爱辉区纳入国家生态文明示范工程试点，在节能减排、循环经济发展等方面获得长久支持，对建设生态黑河，实现永续发展将产生积极而深远的影响。融资渠道进一步拓宽。与国家开行达成战略性合作协议。鼓励金融机构支持地方发展，投放企业贷款81亿元，增长16%，工行、农行、中行、建行联合银团为多宝山铜矿贷款13.2亿元。积极跑省进京，强化对接，向上争取资金66亿元，增长10%。黑河进出口加工产业园纳入省重点，将持续得到省专项资金支持。运用市场化融资模式，筹资5亿元推进园区基础设施建设。扶持首家地方企业鹿源春公司成功上市。招商引资成效显著。创新招商机制，重新制定招商优惠政策和引资人奖励政策，赴京津沪、江浙、深圳和香港等地广泛推介，主动与国内外500强等百余家企业联系沟通，共落实项目184项，到位资金125亿元，增长43%。经积极争取，省政府与俄罗斯6个州区举办的对俄贸易项目推介暨中俄企业合作对接会在黑河召开，聚集了国内和俄罗斯13个联邦主体千余名政界商界代表，开展了多领域洽谈对接，在我省尚属首次，已作为省内最大规模对俄经贸活动机制化落户黑河。成功举办大黑河岛冬季国际经贸洽谈会，分别在黑河和俄布市组织开展洽谈，取得了丰硕成果。发展基础更加坚实。坚持生态发展、绿色崛起不动摇，加入中国绿色碳汇基金会，天保工程二期争取工作得到国家发改委和林业局认可。实施“五年绿化行动”，造林绿化40万亩，森林抚育37.8万亩。生态市建设成效显著，城市环境综合整治考核位居全省前列。“黑河四岛”和黑龙江干流界河国土防护工程得到国家支持，有望争取10亿元建设资金。小型农田水利重点县由北安和嫩江扩大到全市范围，节水灌溉、中小河流治理、水库除险加固等水利工程完成投资5.6亿元，投资规模再创新高。嫩江至黑河公路、前嫩公路伊春至北安段、龙镇至嫩江段交工通车， 尔滨—黑河—漠河航线、北安进京始发列车开通，立体交通网络更加完善。

（二）突出重点育产业，内生动力明显增强。充分发挥资源区位优势，培育特色经济，优化产业结构，“五大产业”成为牵动发展的强力引擎。产业项目拉动强劲。重点产业项目开复工73项，完成投资77亿元，增长30%，开复工率91%，创历史最好水平。26项省重点推进产业项目全部开复工，完成投资54亿元，增长31.7%，超额完成省下达任务。嫩江多宝山铜矿一期、合作区龙江化工10万吨电石、爱辉区酿造储藏一体化酒堡、高纬寒地小浆果种植加工等一批项目投产。现代农业扎实推进。加快调整种植结构，作物面积实现“五增二减”。全市粮食总产80.9亿斤，增长20.9%，首次被国家评为粮食生产先进市。寒地水稻扩种成功，省北部高纬地区推广水稻种植现场会在黑河召开。爱辉区中兴牧业养殖项目奶牛进场，饲料加工项目当年开工、当年投产。冷水鱼养殖规模不断扩大。土地承包经营权加快流转，规模经营面积超千万亩。“三带百村工程”全面启动，局市共建完成“三代”面积810万亩，逊克现代农业示范基地晋升为省级科技示范园区。工业经济提质增效。规模以上工业增加值28.5亿元，增长18%。加快发育战略性新兴产业，爱辉区金湾水电站、小江生物制药等项目加快建设，云计算数据中心、北安上海电气设备制造二期、嫩江凯迪生物质发电等项目开工。扩大传统产业规模，成立市矿业权储备交易中心，集中推进“2335”重点矿业项目，欧林煤矿投产，翠宏山铁多金属矿、争光岩金矿等一批推进多年的矿产开发项目取得突破。建立中小企业服务平台，扶持非公经济发展。对俄合作不断拓展。对俄贸易18亿美元，增长33.8%。整车出口、电力进口翻番，大豆回运实现规模化。阿穆尔—黑河油品储运与炼化综合体、阿州水泥粉磨站、建材综合大市场等项目进展顺利。浮箱固冰通道开通，旅检码头扩建投入使用，输运能力大幅提升。文化旅游加速融合。第三届中俄文化大集上升为国家级文化交流项目，中国国际文化休闲周、边境口岸城市市长论坛、省首届鄂伦春古伦木沓节、五大连池圣水节等一批重大活动，扩大了城市影响力和知名度。“中俄风情歌舞秀”专场演出60余场。五大连池风景区旅游名镇初具规模，火山博物馆开工，机场预可研完成评审。龙江国际交流中心主体完工。接待国内外旅游者486万人次，增长12%。

（三）关注民生增福祉，社会环境和谐稳定。坚持把保障和改善民生作为出发点和落脚点，着力解决群众普遍关心的热点难点问题。城市建设提档升级。坚持“铸魂、提品、增绿、静音”理念，完善城市功能，提升城市形象。深入开展“三城联创”，国家卫生城完成省级申报，园林城基础工作加快推进，环保模范城规划通过环保部评审。全市城建基础设施投资15.2亿元，其中市区2.1亿元。实施了一批硬化、绿化、亮化、美化、净化工程，污水处理厂二期完工，移动式垃圾压缩站在全省首批投入使用。改造旧工业建筑，建成设计理念创新、艺术品位独特的城市规划体验展示馆。城管执法、树木管护和物业管理进社区，城市管理水平进一步提升。社会事业协调发展。实施省级以上科技项目14项，黑河——阿穆尔中俄联合高新区启动前期。全市城镇新增就业2万人。引进紧缺高层次人才36人，外国专家54人次。创建义务教育标准化学校22所，教育质量不断提高。市第一人民医院和市中医院在我省率先实施“先诊疗后结算”服务模式。全市村卫生室均实施了基本药物制度，并实现零差价销售。人口计生工作受到国家表彰。成功承办全国男子武术散打锦标赛、全国男子篮球联赛。社会管理持续加强。城乡社区建设提档升级，爱辉区被评为全国居家养老服务示范区，北安被评为全国村务公开民主管理示范市。全面开展“打非治违”专项行动，安全生产事故起数下降19.4%。重拳打击沙金、煤炭违采盗采行为，维护矿产资源开发秩序。连续13个防期未发生重特大森林火灾。食品药品安全监管工作取得成效。严格执行领导干部接访制度，及时解决群众合理诉求。“平安黑河”建设扎实推进，治安防控体系不断完善。军政军民关系和谐发展，荣获全国双拥模范城“两连冠”，边境秩序持续稳定。防震减灾、老区、侨务、妇女儿童、残疾人、人防、档案、地方史志等工作取得新成绩。

（四）强化服务提效能，政府建设全面加强。主动接受人大依法监督和政协民主监督，173件建议和提案全部办结，民主法制建设不断加强。深入基层调查研究、现场办公，坚持民情恳谈制度，解决群众实际困难。政务公开和办事公开向基层延伸，建立了县级政务公开目录，启动工程建设领域信息公开和诚信体系建设，重点公共企事业单位全面推行办事公开。完善电子政务网络，非涉密文件实现无纸传输。成立市行政服务中心，31个单位168项审批事项集中办理，成为助企便民“政务超市”。推进行政复议试点，组建行政复议委员会专家库，实行专家会诊和集体议决制度。组织大规模应急演练百余次，一般突发事件得到妥善处理。全面推进政府系统廉政风险防控，党风廉政建设不断加强。2012年，我们心系群众，履职践诺，“十二件民生实事”全部完成。“双三零工程”深入推进，30周岁以下高校毕业生就业率85.3%。社保水平持续提升，市直居民基本医疗保险住院医疗费最高支付限额由2万元提高到5.5万元，门诊大病医疗补助最高限额由5000元提高到8000元，低保和优抚群体医疗救助实现“一站式”即时结算。新农合乡镇卫生院住院最高报销比例达到85%以上，报销封顶线由5万元提高到10万元。建立了最低生活保障标准与物价联动机制，保障城乡困难群体生活。高龄补贴扩大到市区所有80周岁以上老年人，失能老人养护中心主体完工。加大教育投入，新建改扩建公办幼儿园28所，创建市级一类幼儿园5所。市区蔬菜储备库交付使用，缓解了冬春淡季蔬菜供应不足。实施饮水安全工程，解决了53 个村屯3.4 万人饮水不安全问题。改善住房条件，全市改造棚户区1.09万户，改造泥草房1.59万户，市区配建廉租房300套。维修老旧住宅楼312栋，受益居民1.5万户。市区输水复线和城市水源地污染防治工程启动。推出东北三省唯一便民自行车服务，设置租赁网点62处，投放自行车1550辆，借还车辆超过400万人次。各位代表，过去一年取得的成绩，得益于市委正确领导，得益于市人大、市政协有效监督和鼎力支持，得益于中省直各单位同心协力和全市广大干部群众团结奋斗。在此，我代表市人民政府，向各级人大代表、政协委员和各民主党派、工商联、无党派人士、人民团体，向关心支持地方工作的中省直单位和人民解放军、武警官兵，向全市人民，表示衷心感谢，并致以崇高敬意！

二、2013 年工作思路目标

2013年是全面贯彻落实党的十八大精神开局之年，是实施“十二五”规划承前启后的关键一年。党的十八大确定了“五位一体”总体布局，提出全面建成小康社会宏伟目标，特别是加大对边疆地区扶持力度，为黑河长远发展提供了广阔空间。俄罗斯入世和加快远东大开发，我国进一步加大沿边开放支持力度，为黑河深化对俄合作带来新的发展机遇。一批重大产业项目建成投产，发展潜力加快释放，为黑河未来发展奠定了坚实基础。须臾懈怠，可能坐失良机；望而却步，或将前功尽弃。只要我们善于运用这些有利条件，充分调动各方面积极性，就一定能够使战略机遇期成为黄金发展期，在追求卓越、跨越赶超的征程中大显身手，大有作为。

政府工作要全面贯彻落实党的十八大和中央、省委经济工作会议精神，坚定实施矿业富市、旅贸活市、科教兴市发展战略，坚持产业项目推动，注重质量效益，提高开放开发层次，扩大投资消费规模，办好民生实事，繁荣边贸旅游明星城，打造特色文化，推动生态保护和绿色发展，促进经济总量与发展质量同步提升，发展速度与幸福指数同步增长，为全面建成小康黑河奠定坚实基础。主要预期目标是：全市生产总值增长12%。全社会固定资产投资增长30%。规模以上工业增加值增长17%。社会消费品零售总额增长15%。公共财政收入增长15%。对外贸易进出口总值增长8%。城镇居民人均可支配收入、农村居民人均纯收入均增长12%。万元地区生产总值综合能耗下降3.2%。

依据黑河欠发达、潜力大的基本市情和发展不足、实力不强、结构不优的阶段性矛盾，今后一个时期，政府工作要坚持强基础、利长远、求创新、促发展的基本方针，牢牢把握好以下几个原则。

——坚持改革开放，激发社会发展活力。突破传统思维定式，强化创新驱动，促进科技与经济紧密结合，扎实推进财政、金融、医疗、文化等重点领域改革和政府运行机制创新，拓展对外开放广度和深度，敢想会干，敢为人先，增强跨越发展的内在活力和持久动力。

——坚持优化布局，促进区域协调发展。树立全市一盘棋思想，依托资源禀赋和发展基础，指导县（市、区）科学确定功能定位和主攻方向，培育特色鲜明、优势突出的主导产业，构建区域错位发展、互补发展、联动发展新格局。

——坚持实业立市，增强经济整体实力。把实体经济作为创造社会财富的重要载体，强化特色园区和产业项目支撑，做优农业、做强工业、做大现代服务业，加快经济结构战略调整，推动产业优化升级，促进工业化、信息化、城镇化、农业现代化同步发展。

——坚持生态优先，全力推进绿色崛起。倍加珍惜良好的生态环境，把生态文明理念贯穿科学发展始终，在开发中保护，在保护中开发，大力发展绿色循环低碳经济，积极培育生态农业、生态旅游业、生物质能源产业，加快构建具有黑河特色的生态主导型产业体系。

——坚持文化引领，提升发展软实力。用文化引领发展、凝聚力量，创新对俄文化交流机制，打造富有地域特点和时代特征的文化品牌，促进边疆文化大发展大繁荣，用文化力提升软实力，用软实力推动硬发展。

——坚持民生为本，共享改革发展成果。集中财力，加大投入，让群众有稳定的工作、满意的收入、舒适的住房，城乡居民享受更优质的医疗服务，孩子们上更好的学校，老人们愉快地安度晚年，让广大群众共享“看得见、摸得着”的幸福。各位代表，新的一年承载新的希望。我们将不断在创新中探索总结，努力在前行中校对坐标，将责任感铭刻在思想上，紧迫感落实到行动上，以更加昂扬向上的精神状态，更加求真务实的工作作风，埋头苦干，勇克难关，把黑河建设得更加美好！

三、打好三大攻坚战实施五大工程，实现经济发展新突破

今年中央经济工作总基调是稳中求进。对黑河而言，稳中求进，进是核心和关键，发展速度必须不断加快。提质增量，量是前提和基础，规模总量必须快速扩张。做好今年经济工作，要找准发展的突破口，集中“三大战役”攻坚，“五大工程”牵动。

（一）打好产业项目攻坚战，提升产业整体竞争力。以项目建设拉动投资快速增长，打牢产业基础，为经济社会发展提供强力支撑。全市安排开复工重点项目118项，年度投资200亿元，增长18%。其中产业项目78项，年度投资105亿元，增长24%。省重点推进产业项目32项，年度投资67亿元，增长35%，开复工率和投资完成率均达到100%。壮大产业集群。优先发展矿产经济，加快发展战略性新兴产业，改造提升传统产业。北安上海电气设备制造二期、爱辉区小江生物制药、合作区龙江化工新增10万吨电石、嫩江燕麦加工、五大连池东谕矿泉饮品等项目建成投产，加快建设爱辉区中兴牧业、云计算数据中心、嫩江铜山铜矿、孙吴精制糖加工、逊克翠宏山铁多金属矿等项目，开工建设嫩江玉米食品加工、爱辉区永发煤矿、北安汽车维修销售中心二期、五大连池圣丰生物育种等项目。增强园区承载能力。完善黑河进出口加工产业园启动区基础设施，采取BT模式融资，投资2亿元建设9条道路、给排水等工程，启动区建成面积达到11平方公里。加快黑河（孙吴）健康产业园建设，引进更多企业，尽快形成规模。争取北安新能源战略产业园纳入省重点。强化财税金融保障。推进部门行政许可与税收征管有机衔接、信息共享，加强地方税费征管和服务。创新管理模式，盘活国有资产存量。积极向上争取政策资金，到位资金增长10%以上。创新金融产品，加大对实体经济支持力度，增强金融机构服务地方经济发展能力。提升小额贷款、融资性担保公司服务质量，扶持更多企业上市。鼓励金融机构支持重点项目和中小企业发展，为企业投放贷款增长15%以上。

（二）打好招商引资攻坚战，实现合作互利共赢。集聚发展要素，开展项目“招商之冬”、“开工之春”活动，以招商大会战助推项目大发展。到位资金增长35%以上。创新招商模式。加强驻外招商，在北京、深圳、哈尔滨等重点区域驻点招商。积极开展以商招商、挂职招商、园区对接招商、科研成果转化招商，努力提高招商实效，在全社会营造浓厚的招商氛围。明确招商责任，政治上给待遇，经济上给保障。招商项目按季度进行集中签约，在新闻媒体通报。发展异地经济。依托各县（市、区）资源优势和主攻方向，打破行政区划界限，建立利益分配机制，联手开展招商引资，合理摆布产业项目，促进资源共享。落实招商引资优惠政策和奖励办法，出台园区优惠政策。加强与国内外友城联系，建立长期合作机制。抓好项目谋划。发挥项目生成研究中心作用，加强与科研院所沟通对接，聘请国内各领域专家，研究谋划一批投资规模大、市场前景好的招商项目。强化市直相关部门招商职能，增设招商科室。研究谋划项目500个以上。扶持非公经济发展。坚持一手抓招商上新项目，一手抓产业链延伸；一手抓大企业大项目，一手抓小微企业发展。实施中小企业成长工程，完善公共服务平台，非公经济增加值增长16%。发挥非公企业主体作用，激发企业家创业活力。对重点企业发放贵宾卡，完善政务环境监测网络，建立跟踪服务机制。提高行政服 务中心运行效率，营造良好发展环境。

（三）打好城市建设攻坚战，打造绿色低碳城市。推进“三城联创”，巩固“三优”成果，促进城市建设与产业发展有序衔接，人与自然和谐相处。坚持规划引领。完成市区城市总体规划修编，与土地利用总体规划、专项规划、园区规划衔接，促进产城互动。扩大城市规模，完善功能分区，构筑“西山、北水、东湿地”城市生态格局，建设“百里界江、万顷湿地、山水环绕、一线多岛”沿江生态长廊，打造以边境旅游、进出口加工、绿色农业、现代服务业为主导的滨江生态口岸城市。加快重点工程建设。着力破解资金、拆迁、征地等瓶颈制约，强力推进市区道路、给排水、供热和公园等重点工程，推动四中北侧路、土产路等道路建设，新建和改建龙江路、开发路、王肃街人行道，打造南部生态植物园，建设污泥处理厂和雨水防洪泵站，完成市区热网改扩建二期和饮用水水源地污染防治工程。鲜花上街，大树进城，利用俄优质树种，实施街路绿化。筹建“俄罗斯城”，建设配套设施完善、俄罗斯风情浓郁的综合性社区。提升城市管理水平。完善现代城市管理体系，建设城市智能化系统，实施网格化管理，提高信息反馈、指挥调度、现场处置能力。抓好城市管理第二轮市场化运作，实行环卫保洁、市政维护、园林管养“三位一体”作业。对官渡路、迎恩路等街路架空管线落地改造，净化城市空间。实施“三优”文明城创建“三进、三延伸”。力争卫生城创建通过国家级评审。丰富城市人文内涵。开展“城市精神”征集活动，凝聚城市力量，铸就城市之魂。提高市民文明素质和礼仪修养，继续开展好“文明过马路、礼让斑马线”等活动，提升城市文明程度，营造和谐友善的城市环境。

（四）实施高纬寒地现代农业示范工程，加快农业现代化步伐。突出标准化、特色化、产业化、市场化，打造高纬寒地农业特色品牌，力争两至三年粮食产能突破百亿斤大关。构建特色农业产业体系。加快调整种植结构，水稻面积增加到40万亩，玉米520万亩。扩大果蔬、蓝莓、木耳等经济作物规模。发展绿色农业，健康安全食品基地达到800万亩以上，农产品质量安全合格率97%以上。筹建爱辉区、五大连池观光农业示范区，开发农事体验和农业文化展示项目。建设现代农业示范园区44个，辐射面积300万亩。加快畜牧业“三带三区”建设。提高冷水鱼养殖效益。扩大中俄林业科技园区繁育基地规模，新建蓝靛果忍冬示范栽培基地1.5万亩。发展北药产业，引进加工企业，种植面积42万亩，增长40%。林业产业产值增长20%。培育新型经营主体。鼓励引导相对成熟的专业合作组织成为经济实体，农民专业合作社发展到860个，其中经营耕地万亩以上的120个，集中打造示范带动能力强的星级社36个。加快土地流转，适宜耕地全部实行规模经营，面积达到1200万亩。推进农业产业化。加快嫩江中药饮片、五大连池华滨面业、孙吴大果沙棘等龙头企业扩建。完善利益共享、风险共担联结机制，产业化基地达到900万亩。建设逊克蓝莓、孙吴果蔬农产品批发市场和龙镇粮食批发交易市场。提高粮食烘干、储运能力，完善粮食综合服务体系。注重研究终端市场，由抓“产供销”向“销供产”转变，把利润更多地留给农民。加强农业基础建设。强化大型现代农机合作社管理，新建水田现代农机合作社3个。执行田间作业标准，农机标准化率由75%提高到80%以上，深松整地500万亩，实现土地科学轮耕。加快小型农田水利重点县和节水增粮行动项目建设，实施旱涝保收田、高效节水灌溉、中小河流治理等工程。争取“黑河四岛”国土防护工程开工。

（五）实施新型城镇化建设工程，促进城乡协调发展。积极稳妥推进城镇化，提高城镇化质量，引导城镇化健康发展，走集约智能、绿色低碳新型城镇化道路。科学合理规划。发挥规划龙头作用，指导各县（市、区）做好城镇规划编制，优化城镇布局，明确发展定位，推动组织实施。实行乡镇整合和迁村进镇，促进近郊村城市化、远郊村城镇化、中心村社区化。将生态文明理念融入城镇化全过程，打造各具特色的小城镇。强化产业支撑。加快培育工业带动型、商贸流通型、旅游发展型、对俄出口型、城郊服务型等城镇经济，推动工业化和城镇化良性互动，城镇化和农业现代化相互协调，不断增强城镇吸纳能力。推进“一乡一业”、“一村一品”，因地制宜发展优势产业，形成特色发展格局。完善基础设施。开展整乡（镇）推进城镇化建设试点，重点打造15个试点乡（镇），经过三至五年努力，完成道路、供热、供水和垃圾处理等基础设施建设，实施美化、净化、亮化和街路两旁绿化，道路硬化率、自来水普及率和住房砖瓦化率均达到100%。加快北安全省城乡一体化整市推进试点。抓好“三带百村”30 个重点村建设。同时，积极探索户籍制度改革，有序推进农业转移人口市民化，逐步实现农民就业、社会保障、管理体制与城市接轨。

（六）实施对俄合作转型升级工程，提高开放型经济水平。推动对俄贸易由速度规模向质量效益转变，由贸易向投资合作、生产加工、园区建设转变，构建优势突出、竞争力强的全方位合作格局。对俄边境贸易增长20%。优化贸易结构。加快对俄贸易转型，培育主营骨干品种，扩大电力、大豆、化工原料等商品进口，增加机电、果蔬、建材及地产品出口，打造出口整车和工程机械重要口岸。加快主体结构转型，培育壮大骨干企业，新增对俄贸易额超亿美元企业2户，加强工程机械、汽车、新材料、农产品等基地培育，力争2家列入省级企业型外贸转型基地，合作区申报省级综合型外贸转型基地。推进重点项目合作。加快投资合作转型，云津集团境外铁矿项目完成签约，阿穆尔—黑河油品储运与炼化综合体具备开工条件，阿州水泥粉磨站开工，建材综合大市场投入使用。支持国际公司对俄电力工程总承包项目建设。加快市场结构转型，逐步扩大俄腹地市场份额。境外机电国际营销网络新建陕汽、中联重科2处网点。争取境外经济技术合作园区纳入省整体规划，完成黑河—阿穆尔中俄联合高新区省级审批和国家级申报，筹建中俄科技企业创业中心，推进跨境经济合作区前期工作。构建便捷顺畅通道。加快口岸物流园区建设，货场投入使用。开工建设旅检气垫船待检场地、浮箱固冰通道道路连接工程。敦促俄方加快黑龙江公路大桥审批，推进铁路大桥前期。五大连池机场核准立项。开展“护企、护商、护游”行动，整治口岸通关秩序，提高通关效率和服务质量。出台互贸区管理办法实施细则，公布互贸区边民携带自用免税进口商品目录。深化对俄合作交流。在“两国一城”框架下，密切与阿州、布市合作，向俄远东腹地城市延伸。加强中俄科技合作，吸引俄高科技人才携技术、项目来我市创业，促进合作成果转化。承办好省对俄贸易项目推介暨中俄企业合作对接会等重大活动，与阿州联办大黑河岛国际经贸洽谈会、阿展会。

（七）实施文化旅游精品工程，放大消费拉动效应。以资源换产业，以产业促增长，打造中俄边境风情游、火山康疗养生游、爱辉历史文化游、森林湿地生态游，建设独具特色的中俄旅游目的地。接待国内外旅游者增长20%。打造精品活动。提升第四届中俄文化大集层次，搭建对俄文化贸易平台。办好首届中俄边境旅游文化节、鄂伦春族下山定居60周年迎庆活动和省第二届古伦木沓节。“中俄风情歌舞秀”演出办出特色、办出水平。将周末大舞台、金色田野等活动打造成群众文化品牌。建设精品景区。加快五大连池旅游名镇建设，开发矿泉温泉，丰富康疗养生产品，综合整治疗养院，改善服务设施。加快格球山景区建设，圣水节前对游人开放。爱辉古城海关历史文化园完成布展设计，孙吴二战遗迹景区陈列馆完成改陈，建设锦河大峡谷休闲旅游度假区。中俄民族风情园、卧牛湖、山口湖、大沾河、大平台雾凇等景区提档升级。策划精品线路。引进知名旅游企业，策划四季旅游产品。开拓俄远东客源市场，抓住俄罗斯“中国旅游年”契机，实施“中俄双子城”旅游规划，开发西伯利亚钻石之旅、贝加尔湖生态之旅、堪察加火山探秘之旅等境外线路。对接省内旅游线路，扩大“情系黑土地”旅游线路影响力，开发黑河—五大连池—伊春、哈尔滨—黑河—漠河黄金旅游线路。整合域内旅游资源，做大漂流游、冰雪游，推出医疗游、自驾游，建设国际旅游养生岛和自驾游营地。与龙广之声等媒体合作，炒热品牌，提升知名度。抓好精品项目。中俄画家村主体完工，打造中俄文化创意产业园、北大荒知青文化产业园。完善博物馆群，加快建设五大连池火山博物馆、北安庆华军工遗址博物馆、中共黑龙江省委和省政府旧址博物馆、嫩江古道驿站博物馆，筹建爱辉新城遗址国家考古公园。开工建设龙江国际文化体育艺术中心和特色酒吧街，龙江国际交流中心投入使用。中俄艺术陈列馆完成改制。同时，完善“吃住行游购娱”配套设施，提高接待和服务水平。

（八）实施森林生态修复工程，全力建设美好家园。加强与大兴安岭、伊春等国有重点林区合作，实施生态建设和修复，共同筑起大小兴安岭绿色生态屏障，为国家生态安全做出贡献。加强生态保护。全力争取纳入天保工程二期。造林绿化39万亩，森林抚育53.5万亩，适宜山区实行封山育林。制定碳汇发展规划，做好碳汇交易前期准备。改善界江沿岸生态环境，建立黑龙江流域沿江岛屿自然保护区，营造界江防护林。筹建生态博物馆。办好黑河中俄林业生态建设学术论坛。推进生态治理。实施“一退三还”行动，重点解决国省道两侧林地“开天窗”问题，恢复逊克干岔子、孙吴红旗等湿地保护区核心区生态，加强草原保护，力争三年内退耕还林还湿还草100万亩。有条件的地区实施生态移民。严厉打击毁林毁湿毁草行为，让青山还树，湿地还水，草原还绿。倡导低碳生活。创建生态文化基地，增强全民生态意识。拓宽公众参与渠道，建立义务植树基地。发展公共交通，倡导绿色出行。提倡低碳建筑，推广环保节能建筑材料。亮化工程采用节能灯具，严格控制光污染。公共机构实行绿色办公、绿色采购。做好节能减排和环境保护工作，加强矿区生态修

复，打造“绿色矿山”。

四、办好十七件民生实事，促进社会和谐稳定回应群众呼声，顺应群众意愿，多谋民生之利，多解民生之忧，在学有所教、劳有所得、病有所医、老有所养、住有所居上投放更多财力，让发展成果实实在在惠及民生。集中办好“十七件民生实事”。努力解决安居问题。（1）全市改造棚户区4664户，配建廉租住房910套。改造农村泥草房1.6万户、危房5000户。推动更高质量就业。（2）实施“双三零工程”，30周岁以下高校毕业生就业率85%以上。全市登记失业率控制在4.5%以内。开展就业援助，发放小额担保贷款6000万元，就业技能培训8000 人次。发展教育卫生事业。（3）市区实施名师名医培养奖励计划，对特殊贡献人员予以重奖，培养、引进和留住高层次人才。（4）全市新建改扩建公办幼儿园16所，创建市级一类幼儿园6 所、标准化学校9所，实施重点部位视频监控全覆盖。加强校园美化、绿化、硬化建设，市直学校全部实现地面硬化，各县（市、区）城内学校两年内实现地面硬化。开展全民健身运动。（5）打造“动感城市”，积极承办重大体育赛事，扶持各类文体协会发展，开展好中小学生上冰雪、万名市民徒步等活动，扩大横渡黑龙江影响力。逐步解决市民冬季健身难问题。办好市第五届体育运动大会。完善社会保障体系。（6）社保扩面提标，城镇职工、居民医疗保险最高支付限额不低于6万元，新型农村社会养老保险新增6万人。建立工伤保险市级调剂金制度。（7）完善城乡医疗救助制度，救助范围从低保对象、五保户扩大到低收入家庭重病、重残等困难群体。取消医疗救助起付线和住院押金，救助封顶线提高到2万元。（8）市区城镇职工、居民基本医疗保险住院医疗费最高支付限额由6万元和5.5万元提高到8万元和6万元。改善市民生活环境。（9）市区建设黑龙江畔浴场，打造群众亲水平台。（10）设立市区应急专项资金200万元，及时解决居民区给排水、供热等突发事件。（11）续建市区输水复线工程，保障供水安全。（12）市区改造小区庭院40个，完善美化、绿化、硬化及文体设施。创新社会管理。（13）爱辉区12个社区全部达到500平方米以上精品社区标准，每个社区辟建1处老年人休闲活动场所。（14）发展老龄事业，全市养老机构增加到60家。办好老年大学。（15）完善民政福利园配套服务功能，建设“三无”老人、失能老人、孤儿和流浪儿童生活“幸福乐园”。（16）市区培训家政服务人员400名，缓解幼儿看护、老人照料等群众关切的市场供需矛盾。（17）开展暖心行动，为市区千名街路保洁员按每人每天6元标准免费提供“阳光早餐”。维护社会安全稳定。高度重视安全生产，完善市县乡三级监管体系，坚决遏制重特大事故发生。加强森林防火工作，确保不发生重特大森林火灾。强化食品安全监督协调组织机构，食品药品检查全覆盖。完善信访联席会议机制，开展好领导干部大接访工作。强化社会治安综合治理，提高综合防控能力，做好社区矫正工作。加强国防后备力量建设，整治界江生产作业秩序，维护边境和谐稳定。同时，支持黑河学院迎接教育部新建院校教学合格评估，进一步做好人口计生、防震减灾、老区、侨务、妇女儿童、残疾人、人防、档案、地方史志等工作。

五、落实中央关于改进工作作风的规定，切实加强政府自身建设全面建成小康社会，实现新的跨越发展，必须提高领导经济工作科学化水平，增强各项工作的科学性、预见性和实效性，努力建设高效率、负责任、有作为的政府。科学决策，依法行政。自觉接受市委领导，主动接受人大、政协监督，高度重视新闻舆论监督和社会监督，完善公众参与、专家咨询、合法性审查、社会稳定风险评估和政府集体议决相结合决策机制。改进作风，完善制度。大兴求真务实之风，深入基层调查研究，及时掌握群众和企业需求。下基层轻车简从、减少陪同、简化接待。精简会议和文件，切实改进会风，开短会、讲短话，力戒空话套话。健全政务公开和办事公开制度，提高工作透明度。狠抓落实，提高效率。推进绩效考核，强化过程控制，严厉治庸、治懒、治散，坚决纠正行政不作为和乱作为问题。认定的事一抓到底，承诺的事全部兑现，确保令行禁止、政令畅通。勤俭节约，清正廉洁。全面落实政府系统廉政风险防控工作，规范权力运行。严格控制部门预算，压缩一般性开支，节约行政成本。加强政府投资项目审计监督，规范投资行为。强化效能监察，打造廉洁高效的政务环境。各位代表，2012是不同凡响的一年，我们风雨兼程，脚步坚定；2013承载着全面建成小康社会的美好愿望，我们激情满怀，乘势启航。让我们在市委坚强领导下，紧紧依靠全市人民，凝聚起共同奋斗的智慧和力量，以攻坚克难的勇气和担当，全面完成各项目标任务，谱写黑河跨越发展的新篇章！
